# Risk-Return Analysis of the Biopharmaceutical Industry as Compared to Other Industries

**DOI:** 10.3389/fphar.2018.01108

**Published:** 2018-10-01

**Authors:** Cedric Popa, Karel Holvoet, Tessa Van Montfort, Floris Groeneveld, Steven Simoens

**Affiliations:** ^1^Deloitte Belgium, Zaventem, Belgium; ^2^Department of Pharmaceutical and Pharmacological Sciences, KU Leuven, Leuven, Belgium

**Keywords:** biopharmaceutical industry, profitability, risk, return, risk-adjusted return on investment

## Abstract

**Background:** Profits in the biopharmaceutical industry have been scrutinized in social debate. However, drawing conclusions based on industry profitability only is inappropriate as such an analysis does not account for risks faced by investors. This study aims to measure risks and returns in the biopharmaceutical industry and investigates whether risk-adjusted return on investment in the biopharmaceutical industry is higher than that in other industries.

**Methods:** To enable appropriate comparison, we identified six benchmark industries with characteristics that match those of the biopharmaceutical industry: automotive manufacturing, commercial aircraft manufacturing, consumer electronics, packaged food manufacturing, telecom, and oil and gas. For those industries, we selected the top 25 companies per industry, covering 35–65% of industry revenues. Data on return measures (i.e., net profit margin, return on equity, total shareholder return) and risk measures (i.e., volatility of total shareholder return, beta) were derived from Bloomberg over the 2004–2016 period. The Sharpe ratio was calculated as a measure of risk-adjusted return on investment and compared between industries.

**Results:** Net profit margins varied between 12.6 and 19.5% in the biopharmaceutical industry, and ranged from 2.6 to 8.4% in the benchmark industries. Return on equity for the biopharmaceutical industry was above the average for the other industries. Total shareholder returns for the biopharmaceutical industry amounted to 11.7%, ranking fifth across the seven industries. The biopharmaceutical industry ranked sixth among the seven industries regarding beta, and sixth in terms of volatility of total shareholder return. The median Sharpe value for the biopharmaceutical industry ranked fifth of seven industries.

**Conclusion:** Over the 2004–2016 period, the biopharmaceutical industry did not attain risk-adjusted return on investment in excess of that in other industries and, thus, did not outperform these industries.

## Introduction

The biopharmaceutical industry is facing changing–and challenging–times (Gronde et al., 2017): increasingly stringent regulations in the pharmaceutical and healthcare area, development costs for pharmaceutical compounds increasing significantly, and an intensifying search for new, value-based payment models. At the same time, most governments are struggling to balance health and social expenditures at sustainable levels.

In this context, prices and profits in the biopharmaceutical industry have been increasingly scrutinized in social debate (McCarthy, 2015; Kesselheim et al., 2016; Sibbald, 2017). In our opinion, drawing conclusions based on industry profitability only is inappropriate for at least two reasons. First, return on investment needs to be measured, as high profits do not automatically translate in high investor returns. Second, given that risk-taking is an integral part of investing and doing business, return on investment needs to be evaluated in light of the risks faced by industry participants and investors. However, to date, little is known about risks and returns of the biopharmaceutical industry.

The aim of this study is to investigate whether risk-adjusted return on investment in the biopharmaceutical industry is higher than that in other industries. To this effect, this study measures risks and returns in the biopharmaceutical industry, compares these indicators with comparable industries involved in the development of innovative products, and investigates the level of risk-adjusted return on investment for the biopharmaceutical industry as compared to these other industries.

## Materials and Methods

### Selection of Comparator Industries

According to the Bloomberg Industry Classification System (BICS) (Bloomberg, 2014), more than 60 industries are recognized as distinct from one another. To enable appropriate comparison, we identified benchmark industries with characteristics that match those of the biopharmaceutical industry. The following characteristics were used: (a) marketing of well-defined, tangible products or services that are quantifiable in a metric; (b) level of research and development (R&D) investment; (c) level of industry maturity; and (d) degree of market concentration and delineation of each sector. Based on this analysis, six industries were chosen as comparable with the biopharmaceutical industry: automotive manufacturing, commercial aircraft manufacturing, consumer electronics, packaged food manufacturing, telecom, and oil and gas. For those industries, we selected the top 25 companies per industry, covering 35–65% of industry revenues.

### Data

Data were extracted from Bloomberg, given that this is a clear and replicable data source. The primary dataset included data on sales, operating and net profit margin, R&D costs, equity, capital employed, stock prices, and betas.

### Risk and Return Measures

With respect to return measures, the ability to generate profit is a key determinant of a company’s performance. Consequently, we applied a commonly used return measure, the net profit margin. This measure is calculated as net income divided by sales. Given that the net profit margin is an indicator of certain aspects of return, we also calculated a more comprehensive metric, return on equity, which combines several financial and operating efficiency measures. The return on equity metric is the product of the net profit margin, asset turnover (i.e., ratio of sales to total assets) and financial leverage (i.e., ratio of total assets to equity investments). A third return measure was computed, namely total shareholder return, which is defined as the return to shareholders from both dividends and from appreciation of the underlying investment.

Another issue of social debate relates to the industry spend on R&D. Therefore, we compared R&D expenses as a percentage of gross margin and as a percentage of sales across the selected industries.

In terms of risk measures, we distinguished between business/operational risk faced by industry participants and risks faced by investors. The former was documented by calculating year-on-year changes in net profit margins and by calculating the inter–quartile range as a measure for dispersion of net profit margins across companies within an industry. The latter was exemplified by means of two widely used risk measures, volatility of total shareholder return and correlation of specific investment returns with market returns (beta). We calculated the weekly volatility in shareholder return and the 2-year weekly beta of each company.

The Sharpe ratio was used given that this is a widely accepted measure of risk-adjusted return on investment. This ratio is calculated as the excess total shareholder return over the risk-free rate divided by the stock’s volatility.

### Time Horizon

The analysis was based on a 12-year historical period from 2004 to 2016. This extended time horizon has the advantage of capturing industry trends across both downward and upward fluctuations of the economic cycle.

### Data Analysis

A descriptive analysis was carried out, calculating return, R&D and risk measures for each company in each industry, and comparing median values of these measures across multiple years between industries.

## Results

**FIGURE 1:**
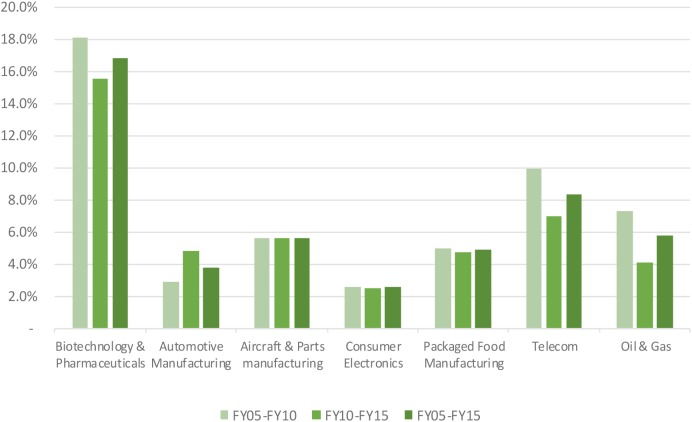
Net profit margin of selected industries.

**FIGURE 2:**
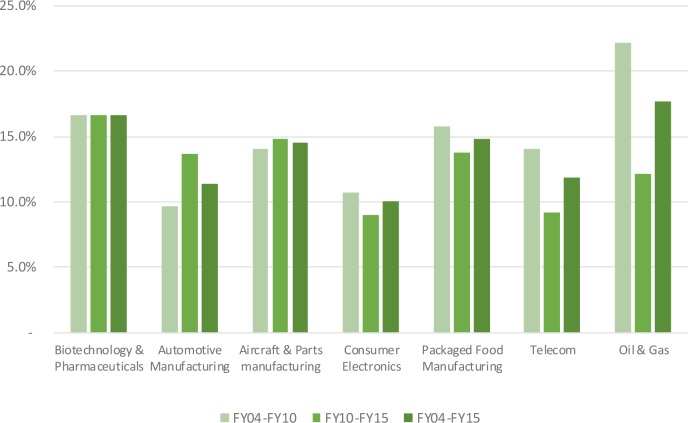
Return on equity of selected industries.

**FIGURE 3:**
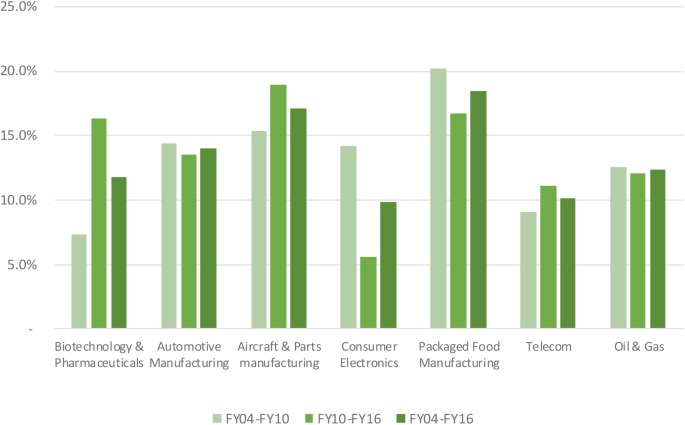
Total shareholder return of selected industries.

Median net profit margins in the biopharmaceutical industry fluctuated around 16.8% level, reaching a maximum level of 19.5% in 2009 and a minimum level 12.6% in 2014 (see **Figure 1**). Net profit margins ranged from 2.6 to 8.4% for the benchmark industries. In addition to the net profit margin, other return indicators include asset turnover and financial leverage. Asset turnover in the biopharmaceutical industry was relatively stable over time at a 53% level, and was consistently and substantially below the benchmark industries (with the exception of the telecom sector). The financial leverage ratio for the biopharmaceutical industry was two, ranking the industry the lowest compared to the benchmark industries. These three metrics were combined to generate the return on equity for the biopharmaceutical industry, which ranked amongst the highest, slightly above returns on equity in the aircraft and parts manufacturing and packaged food manufacturing (see **Figure 2**). Finally, **Figure 3** shows that total shareholder returns over the 2004–2016 period for the biopharmaceutical industry amounted to 11.7%, ranking fifth across the seven industries included in this study.

**FIGURE 4:**
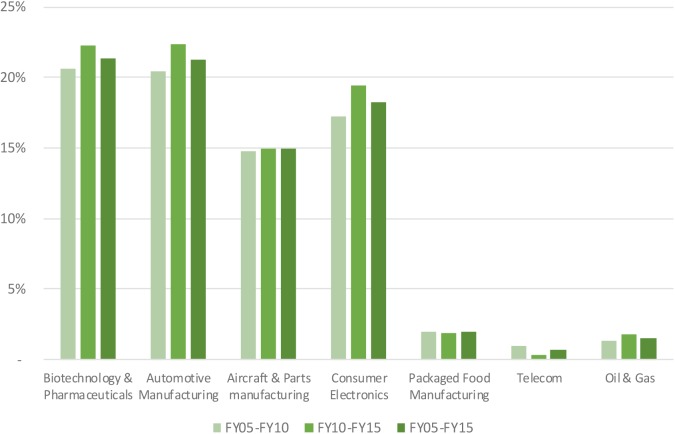
R&D expenses as percentage of gross margin of selected industries.

Research and development expenses as a percentage of gross margin amounted to 21% for the biopharmaceutical industry (see **Figure 4**). This percentage was in line with that of the automotive industry and above that of the other benchmark industries. The biopharmaceutical industry reinvested 16% of sales back into R&D, with the consumer electronics sector being the second most R&D-intense industry at 5%.

**FIGURE 5:**
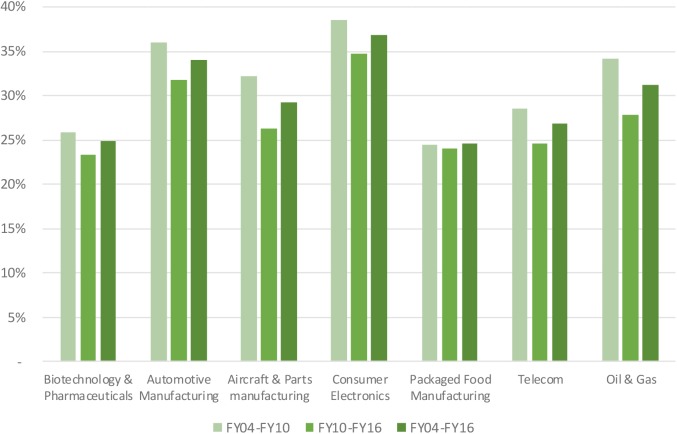
Volatility of total shareholder return of selected industries.

**FIGURE 6:**
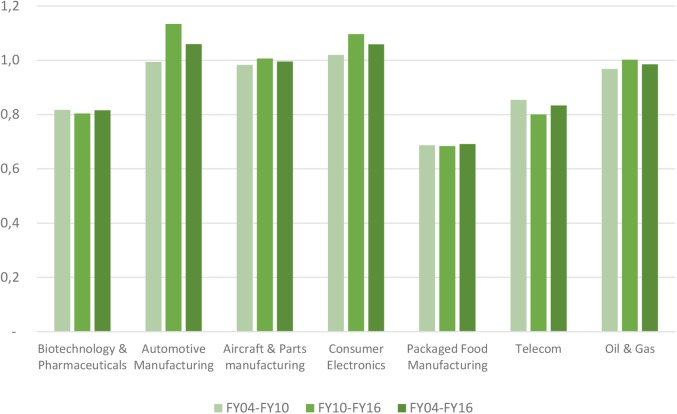
Two-yearly beta of selected industries.

With respect to business/operational risk faced by industry participants, the biopharmaceutical industry exhibited a volatility in net profit margins twice as high as the benchmark industries (median standard deviation of 9.3% in the biopharmaceutical industry, 4.9% in the packaged food industry or below for the other industries). The biopharmaceutical industry had the highest intra-industry dispersion of net profit margins, with an inter-quartile range of 12.5%, which exceeded that of the benchmark industries (inter–quartile ranges between 3.7% for the automotive industry and 7.9% for the telecom sector). With respect to risks faced by investors, the biopharmaceutical industry ranked sixth among the seven industries in terms of volatility of total shareholder return (see **Figure 5**) and also ranked sixth in terms of beta (see **Figure 6**).

**FIGURE 7:**
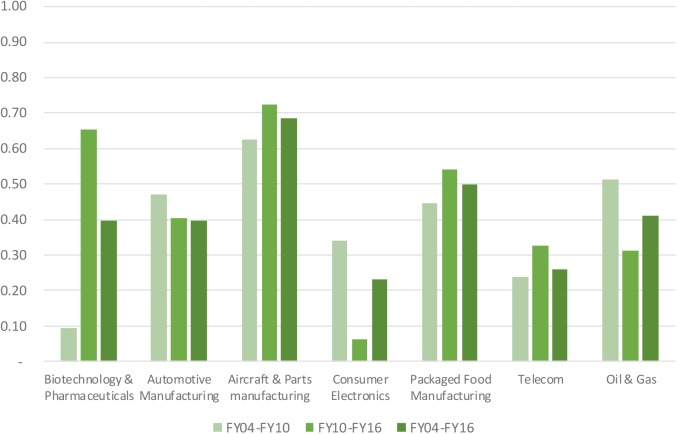
Sharpe ratio of selected industries.

**Figure 7** compares the median Sharpe ratio across the selected industries from 2004 to 2016. The median Sharpe ratio for the biopharmaceutical industry ranked fifth out of the seven industries in terms of risk-adjusted performance.

## Discussion

Our return analysis showed that the biopharmaceutical industry outperformed comparator industries in terms of net profit margins. When we used a standard return measure in financial analysis, return on equity in the biopharmaceutical industry was above the average for the benchmark industries (with differences being much lower than differences observed in net profit margins). However, our analysis of total shareholder returns indicated that investors in the biopharmaceutical industry have not realized returns that outperform those of other industries. Compared to the benchmark industries, the biopharmaceutical industry was first-in-class in terms of spending on R&D (as a percentage of gross margin and of sales).

Our analysis suggested that the biopharmaceutical industry faces higher business/operational risk than the benchmark industries. This was exemplified by the observations that intra-industry dispersion of net profit margins and volatility of net profit margins over time are larger within the biopharmaceutical industry than within the benchmark industries. Also, the projected returns for R&D investments demonstrated a year-on-year downward trend since 2010. The finding of higher business/operational risk may arise from a number of reasons: (a) product development time is the longest in the biopharmaceutical industry; (b) regulation of the biopharmaceutical industry is amongst the highest, most complex, and most fragmented; (c) the effective period of patent protection is lower than for other industries; and (d) the biopharmaceutical industry (along with the food and automotive industries) appears to be more competitive than the other industries.

However, data on volatility of total shareholder return and on beta showed that risks faced by investors in the biopharmaceutical industry are not substantially higher than those faced by investors in any of the benchmark industries. This suggests that, although investors face risks specific to the biopharmaceutical industry (associated with developing risky R&D-intensive assets through the pipeline as well as exposure to changes in third-party reimbursement, pricing regulations of medicines, etc.), investors do not receive any return for accepting them, as these risks can be diversified away by forming a portfolio of assets that are not correlated. Also, we believe that the structure of the biopharmaceutical industry acts as a mitigating factor for these specific risks, given that the biopharmaceutical industry benefits from patent protection, market exclusivity and premium pricing for bringing innovative medicines to the market.

Our study also linked risks faced by investors to returns. To this effect, we used the Sharpe ratio, which describes how much excess return an investor is receiving for the extra volatility that the investor endures for holding a riskier asset. An advantage of this approach is the wide acceptance of these risk and return measures. Furthermore, the study drew on observable and market-derived data and hence provided insight into how financial markets value business performance considering the level of market risk assumed in the industry. Our findings showed that total risk-adjusted returns for the biopharmaceutical industry were very close to the average of the benchmark industries. In other words, over the last 12 years, investors in the biopharmaceutical industry have not realized risk-adjusted returns in excess of those in the benchmark industries.

To the best of the authors’ knowledge, only one study has previously been published that rigorously examined risk-return measures within and across industries (Thakor et al., 2017). However, this study primarily contrasted pharmaceutical companies with biotechnology companies, whereas the focus of our analysis was to compare the whole biopharmaceutical industry with other industries. Also, this study was restricted to publicly traded United States biopharmaceutical companies, whereas our analysis did not apply such an inclusion criterion.

Our study is subject to a number of limitations. First, our study covered an extensive historical period of 12 years and contrasted the biopharmaceutical industry with six other industries selected according to a set of criteria. An extension of the study to a longer historical period and to a higher number of industries may provide additional insights. However, the choice for a longer historical period needs to be balanced against issues such as the relevance of “old” data to the current economic climate, changes in the basket of top 25 companies per industry over time, and survival bias. Also, if more industries are included that are less comparable, the cross-industry comparisons become less relevant.

Second, a potential limitation is that return measures may depend on technical aspects of the accounting standards used (e.g., R&D capitalization, acquisition accounting, etc.). Companies have latitude when choosing certain accounting methods. Ratios taken from financial statements that employ different accounting choices may not be entirely comparable, mostly when performing cross-industry comparisons. R&D-intensive industries are especially exposed, as the accounting treatment of R&D spend is heavily dependent on judgement in combination with different accounting principles.

Third, we acknowledge that the risk profile of R&D assets changes when it is moved through the R&D pipeline to market. However, our study included large companies that hold R&D assets in all stages of the R&D cycle (from early stage to market). As such, the resulting risk profile reflects the risk for sector participants with a diversified portfolio of R&D assets. Also, financial information is typically available only at the level of the company (as opposed to linked to specific products).

Fourth, the study did not differentiate between sub-sectors of the biopharmaceutical industry (e.g., vaccines, orphan medicines, off-patent medicines). However, an extended analysis would only be possible if there are quoted companies focused on such sub-sectors. For instance, for the largest vaccine companies, vaccines still represent only 10–15% of their total sales, hence it is not possible to conduct a risk-return analysis for sub-sectors.

## Data Availability Statement

The data analyzed in this study were obtained from Bloomberg.

## Author Contributions

SS and CP conceived and designed the study in consultation with all authors. CP, KH, TVM, and FG were involved in the data collection, data analysis, writing the report, and revised the draft manuscript. SS wrote the draft manuscript. All authors collaborated in this study, interpreted the results, and approved the final version.

## Conflict of Interest Statement

CP, KH, TVM, and FG are employees of Deloitte and SS is an employee of KU Leuven. Deloitte in collaboration with KU Leuven received an unconditional grant from GSK and Janssen Cilag to carry out this study.
